# Quality improvements of healthcare trajectories by learning from aggregated patient-reported outcomes: a mixed-methods systematic literature review

**DOI:** 10.1186/s12961-022-00893-4

**Published:** 2022-08-17

**Authors:** Maarten C. Dorr, K. S. van Hof, J. G. M. Jelsma, E. A. C. Dronkers, R. J. Baatenburg de Jong, M. P. J. Offerman, M. C. de Bruijne

**Affiliations:** 1 Department of Otorhinolaryngology and Head and Neck Surgery, Erasmus MC Cancer Institute, Erasmus University Medical Center, Dr. Molewaterplein 40, 3015 GD Rotterdam, The Netherlands; 2 Department of Public and Occupational Health, Amsterdam Public Health Research Institute, Amsterdam UMC, Vrije Universiteit Amsterdam, Van der Boechorststraat 7, 1081 BT Amsterdam, The Netherlands

**Keywords:** Value-based healthcare, Patient-reported outcome measures, Quality improvement, Aggregated level, Benchmarking

## Abstract

**Background:**

In healthcare, analysing patient-reported outcome measures (PROMs) on an aggregated level can improve and regulate healthcare for specific patient populations (meso level). This mixed-methods systematic review aimed to summarize and describe the effectiveness of quality improvement methods based on aggregated PROMs. Additionally, it aimed to describe barriers, facilitators and lessons learned when using these quality improvement methods.

**Methods:**

A mixed-methods systematic review was conducted. Embase, MEDLINE, CINAHL and the Cochrane Library were searched for studies that described, implemented or evaluated a quality improvement method based on aggregated PROMs in the curative hospital setting. Quality assessment was conducted via the Mixed Methods Appraisal Tool. Quantitative data were synthesized into a narrative summary of the characteristics and findings. For the qualitative analysis, a thematic synthesis was conducted.

**Results:**

From 2360 unique search records, 13 quantitative and three qualitative studies were included. Four quality improvement methods were identified: benchmarking, plan-do-study-act cycle, dashboards and internal statistical analysis. Five studies reported on the effectiveness of the use of aggregated PROMs, of which four identified no effect and one a positive effect. The qualitative analysis identified the following themes for facilitators and barriers: (1) conceptual (i.e. stakeholders, subjectivity of PROMs, aligning PROMs with clinical data, PROMs versus patient-reported experience measures [PREMs]); (2a) methodological—data collection (i.e. choice, timing, response rate and focus); (2b) methodological—data processing (i.e. representativeness, responsibility, case-mix control, interpretation); (3) practical (i.e. resources).

**Conclusion:**

The results showed little to no effect of quality improvement methods based on aggregated PROMs, but more empirical research is needed to investigate different quality improvement methods. A shared stakeholder vision, selection of PROMs, timing of measurement and feedback, information on interpretation of data, reduction of missing data, and resources for data collection and feedback infrastructure are important to consider when implementing and evaluating quality improvement methods in future research.

**Supplementary Information:**

The online version contains supplementary material available at 10.1186/s12961-022-00893-4.

## Key messages

**What is already known on this topic**

The aggregated patient-reported outcome measures (PROMs) can be used for analytical and organizational aspects of improving and regulating healthcare, but there is little empirical evidence regarding the effectiveness of aggregated PROMS.

**What this study adds**

This study adds a detailed overview of the types of quality improvement methods and recommendations for implementation in practice.

**How this study might affect research, practice or policy**

Researchers and policy-makers should consider the barriers, facilitators and lessons learned for future implementation and evaluation of quality improvement methods, as presented in this manuscript, to further advance this field.

**Supplementary Information:**

The online version contains supplementary material available at 10.1186/s12961-022-00893-4.

## Background

Since the introduction of value-based healthcare by Porter [1] in 2006, an increase in the use of patients’ perspectives on health outcomes for quality and safety improvement in healthcare has been observed [2], in addition to process and clinical outcomes [3–5]. These so-called patient-reported outcome measures (PROMs) capture a person’s perception of their own health through standardized, validated questionnaires [6]. The main purpose of PROMs is to improve quality of care and provide more patient-centred care by quantifying important subjective outcomes, such as perceived quality of life and physical and psychosocial functioning.

For the purpose of quality improvement in healthcare, PROMs are used on a micro, meso and macro level. On a micro level, PROMs are useful screening and monitoring tools to facilitate shared decision-making and patient-centred care [7–9]. On a meso level, aggregated PROMs (i.e. PROM outcomes on the group level) provide analytical and organizational angles for improving and regulating health in specific populations as a result of enhanced understanding, self-reflection, benchmarking and comparison between healthcare professionals and practices [10–12]. At a macro level, PROMs are used for overall population surveillance and policy [2, 13, 14]. The use of structurally collected PROMs is increasingly adopted in national quality registries [15, 16], and it increased even further after the Organisation for Economic Co-operation and Development (OECD) recommended the collection of aggregated PROMs to obtain insight into system performance and to enable comparative analysis between practices [17].

The use of aggregated PROMs is a relatively young field. In 2018, Greenhalgh et al. showed that there was little empirical evidence that PROMs, at a meso level, led to sustained improvements in quality of care [18]. However, since then, there has been growing interest in this field, with a plethora of quantitative and qualitative research currently available. Therefore, the aim of this mixed-methods systematic review was threefold: (1) to summarize quality improvement methods based on aggregated PROMs at the meso level in hospital care; (2) to describe the effectiveness of quality improvement methods; and (3) to describe barriers, facilitators and lessons learned when using aggregated PROMs for quality improvement in healthcare.

## Methods

The Preferred Reporting Items for Systematic Reviews and Meta-Analyses (PRISMA) guidelines were used to design and report this review [19]. The review was prospectively registered with the International Prospective Register of Systematic Reviews (PROSPERO) 07-12-2020 (PROSPERO 2020: CRD42020219408).

### Search strategy

Embase, MEDLINE, CINAHL and the Cochrane Library were searched for studies published up to May 2021. The search strategy (Additional file 1: Appendix I) included terms related to outcome measurements, quality management and quality improvement. Search terms consisted of Medical Subject Headings (MeSH) and free-text words, wherein for most terms, synonyms and closely related words were included. The search was performed without date or language restriction. Additional references were obtained by hand-searching reference lists of included studies and systematic reviews (backwards selection) and by identifying studies that cited the original included studies (forward selection). Duplicate studies were removed.

### Eligibility criteria

Studies were considered eligible for inclusion if they described, implemented or evaluated a quality improvement method based on aggregated PROMs in the curative hospital setting. Both quantitative and qualitative studies were included in this review. Quantitative studies included experimental study designs, such as randomized controlled trials, controlled trials, cluster trials, controlled before–after studies and time-series studies. Qualitative studies included semi-structured interviews, focus groups or studies with a mixed-methods approach (e.g. process evaluation studies). Studies were excluded for the following: (1) the quality improvement was based on the use of PROMs in the individual setting only (e.g. in the consultation room); (2) written in a language other than English; (3) not peer-reviewed; (4) conference and editorial papers and reviews; or (5) the full text could not be obtained.

### Study selection

All records found were uploaded to Rayyan, an online web application that supports independent selection of abstracts [20]. Two researchers (KvH and MD) independently screened the titles and abstracts of the identified studies for eligibility. Discrepancies were resolved by discussion with the involvement of a third researcher (JJ) when necessary. Subsequently, full texts were screened against the eligibility criteria independently by two researchers (KvH and MD).

### Data extraction and synthesis

Due to the mixed-methods design of this review, two researchers (KvH and MD) extracted data from qualitative and quantitative studies separately [21] using a standardized form. Details on the study design, aims, setting, sample size, quality improvement method, PROMs and outcomes were extracted and synthesized into a narrative summary. The described quality improvement methods were summarized, and when available, the effect of these methods was reported.

For the qualitative synthesis, the approach outlined by Thomas and Harden [22] was followed, which involved a thematic synthesis in the form of three stages: (1) free line-by-line coding of the findings performed by three researchers; (2) organization of these codes into related areas to construct descriptive themes; and (3) the development of analytical themes. A fourth researcher (MO) was consulted for verification and consensus. The qualitative synthesis was structured around facilitators, barriers and lessons learned for the implementation of quality improvement interventions based on PROM data. Finally, both quantitative and qualitative synthesis were combined in the discussion section.

### Quality assessment

Study quality was assessed independently by two researchers (KvH and MD) with the validated Mixed Methods Appraisal Tool (MMAT) [23] informing the interpretation of findings rather than determining study eligibility. The MMAT is a critical appraisal tool that is designed for mixed-methods systematic reviews and permits us to appraise the methodological quality of five study designs: qualitative research, randomized studies, non-randomized studies, descriptive studies and mixed-methods studies. Aspects covered included (dependent on study design) quality of study design, randomization, blinding, selection bias, confounding, adherence and completeness of data. The MMAT does not provide a threshold for the acceptability of the quality of the studies [23].

## Results

**Fig. 1:**
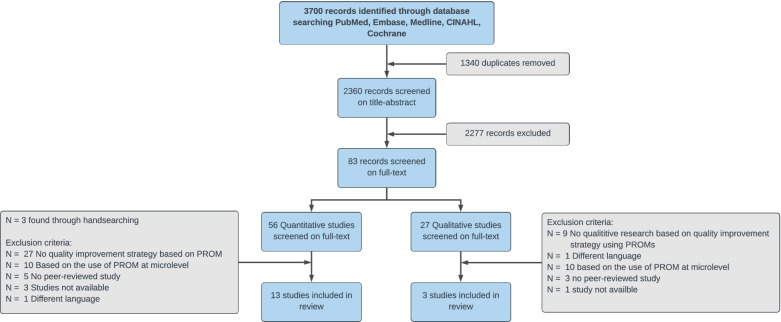
Flow diagram of the search process and study selection

A flow diagram of the study selection process is presented in Fig. 1. A total of 3700 records were identified. After removing duplicates, 2360 records were screened on title–abstract, and 83 records were screened on full text. Three studies were found through hand searching [24–26]. Finally, 13 quantitative studies [24, 25, 27–36] and three qualitative studies [10, 11, 37] met the inclusion criteria. Research questions 1 and 2 are addressed in the “Quantitative studies” section, and research question 3 is addressed in the “Qualitative studies” section.

### Quality of the studies

The quality assessment was performed according to study design: quantitative randomized [24, 28], quantitative non-randomized [25–27, 29, 30, 33, 34, 36], quantitative descriptive [31, 32, 35] and qualitative studies [10, 11, 37]. Five studies were assessed as good-quality studies, and the other 11 were assessed as moderate-quality studies. Neither randomized study was able to blind healthcare professionals to the intervention provided, although since receipt or non-receipt of feedback in these studies could not be disguised, this was not weighed as poor quality. Lack of complete outcome data was a shortcoming in five of the studies [24, 26, 29, 30, 33, 34]. In addition, for two descriptive studies [31, 35], it was not possible to assess response bias. The quality assessment can be found in Additional file 2: Appendix II.

## Quantitative studies

### Study characteristics

Table 1 summarizes the study characteristics of the 13 included quantitative papers. The search yielded two randomized controlled trials [24, 28], eight non-randomized controlled studies [25–27, 29, 30, 33, 34, 36] and three single-centre descriptive studies [31, 32, 35]. Studies were performed in the United States [24, 26, 27, 35], United Kingdom [30, 32, 34], Netherlands [25, 33], Sweden [31], Denmark [29], Canada [36] and Ireland [28]. Twelve studies focused on patients from surgical specialties, including orthopaedic [26, 28, 30, 32, 35], thoracic [29, 33], urologic [27, 36], ophthalmic [31], rhinoplastic [25] and general surgery [34]. One study focused on primary care [24]. In eight studies, data were obtained from a regional or national quality registry [27, 29–35]. The included studies used generic PROMs [30, 33], disease-specific PROMs [25, 27, 29, 31] or a combination of generic and specific PROMs [24, 26, 28, 32, 34–36].

**Table 1:** Study characteristics, quality improvement methods and/or outcome

References	Aim	Design	Setting	PROM	Quality improvement method	Outcome
Boyce et al. [28]	To assess whether peer-benchmarked feedback is effective in improving patient outcomes	Randomized	Orthopaedics *N* = 21 surgeons Ireland	Oxford Hip Score (DS) Hip Osteoarthritis Outcome Score (DS) EuroQol 5D (G)	Peer-benchmarked feedback and educational intervention	(−) No effect from peer-benchmarked feedback was found on patient-reported outcomes
Weingarten et al. [24]	To determine whether providing physicians with peer-comparison feedback can improve patient functional status	Randomized	Primary care *N* = 48 surgeons United States	Dartmouth Primary Care Cooperative Information Project chart	Peer-comparison feedback and educational intervention	(−) No improvement in patient functional status
Varagunam et al. [34]	To determine the impact of introduction of PROMs on the selection of patients and on outcome	Non-randomized	General surgery *N* = 409 surgeons United Kingdom	Oxford Hip Score (DS) Oxford Knee Score (DS) Aberdeen varicose vein questionnaire (DS) EuroQol 5D	Peer-benchmarked feedback	(±) No to minimal impact of routine use and feedback of PROMs was found
Kumar et al. [36]	To determine whether providing surgical report cards to surgeons resulted in improved patient outcomes	Non-randomized	Urologic surgery *N* = 8 surgeons Canada	Expanded Prostate Cancer Index Composite (DS) EuroQol 5D (G)	Peer-benchmarked feedback	(−) No improvement in functional or oncologic outcomes
Van Veghel et al. [33]	To assess patient-relevant outcomes of delivered cardiovascular care, to establish and share best practices by comparing outcomes and to embed value-based decision-making to improve quality and efficiency	Non-randomized	Cardiac surgery *N* = 12 centres Netherlands	Short Form Health Survey 36 (G) Short Form Health Survey 12 (G)	PDSA cycle and benchmarking	Not applicable
Bronserüd et al. [29]	To propose a model for the use of PROMs as quality indicators, enabling comparison across surgical departments	Non-randomized	Thoracic surgery *N* = 4 departments Denmark	EORTC-QLQ-C30 (DS)	Benchmarking surgical departments	Not applicable
Van Zijl et al. [25]	To present a method to measure and evaluate data-based performance	Non-randomized	Rhinologic surgery *N* = 1 surgeon Netherlands	Nasal Obstruction Symptom Evaluation (DS) Utrecht Questionnaire (DS) Visual analogue scale (DS)	Information technology (IT) application Dashboarding	Not applicable
Reilly et al. [26]	To develop a novel approach to consistently and pragmatically measure the value of total knee and hip arthroplasty	Non-randomized	Orthopaedics *N* = 6 surgeons	Physical function domain from the PROMIS-10 (G) Hip Osteoarthritis Outcome Score (DS) Knee Osteoarthritis Outcome Score (DS)	IT application Dashboarding	Not applicable
Lucas et al. [27]	To report on the establishment of a web-based collection system and measure variability in outcome among practice groups	Non-randomized	Urologic surgery *N* > 40 centres United States	Symptom Tracking and Reporting (DS)	Benchmarked reports for individual surgeons	Not applicable
Gutacker et al. 2013 [30]	To measure the extent to which treatment impact varies across hospitals	Non-randomized	Orthopaedics *N* > 153 hospitals United Kingdom	EuroQol 5D (G)	Hospital benchmarking	Not applicable
Partridge et al. [32]	To improve PROMs after implementation of evidence-based change in practice	Descriptive	Orthopaedics *N* = 14 surgeons United Kingdom	Oxford Knee Score (DS) EuroQol 5D (G)	PDSA cycle	(+) Significant improvement on the Oxford Knee Score and EQ-5D
Lundström et al. [31]	To analyse three models enabling data connection between PROMs and clinical data in order to identify opportunities for improvement of quality of care	Descriptive	Ophthalmology *N* = 41 surgeons Sweden	Catquest-9SF (DS)	Aggregated internal analyses	Not applicable
Zheng et al. [35]	To present the design and implementation of a website which is able to return comparative patient-reported outcome reports for participating surgeons in order to monitor and improve quality and health outcomes	Descriptive	Orthopaedics *N* > 130 surgeons United States	Short Form Health Survey 36 (G) Knee Injury and Osteoarthritis Outcome Score (DS)	Site, practice and individual benchmarking	Not applicable

*G* general measurement instrument, *DS* disease-specific measurement instrument, *PDSA* plan-do-study-act cycle, *EORTC-QLQ-C30* European Organisation for the Research and Treatment of Cancer Quality of Life Questionnaire, *PROMIS* Patient-Reported Outcomes Measurement Information System

### Effect and impact

Only five out of 13 studies reported on the effect of quality improvement methods based on aggregated PROMs [24, 28, 32, 34, 36]. Four of these studies, including both randomized controlled trials, showed no effect [24, 28, 36] to a minimal effect [34] on patient-reported outcomes after the use of individual benchmarking as a quality improvement method (Table 1). One of the studies showed a significant improvement in the Oxford Knee Score after a plan-do-study-act (PDSA) cycle in a cross-sectional post-intervention cohort [32]. The other eight studies described the method of implementation without effect measurement [25, 27, 33, 35], or discussed (statistical) models for using aggregated outcomes as performance indicators [29–31].

### Methods used to accomplish quality improvements

Four quality improvement methods were identified: benchmarking [24, 27–30, 34–36], PDSA cycles [32, 33], dashboards as feedback tool [25, 26] and internal statistical analysis [31] (Table 2).

**Table 2:** Quality improvement methods

Quality improvement method	Aim
Benchmarking	Method in which PROMs are used by departments or individual healthcare professionals to compare their own performance with peers in order to improve their performance
Plan-do-study-act (PDSA) cycle	An iterative, four-stage problem-solving model used for improving a process
Dashboard as feedback tool	A dashboard summarizes and visualizes data. It enables monitoring and managing of performance outcomes
Aggregated statistical analysis	Use of data analysis in order to identify opportunities for quality improvement

### Benchmarking

Benchmarking was applied in eight studies [24, 27–30, 34–36]. Aggregated data were used to provide peer-benchmarked feedback for individual healthcare professionals [24, 27, 28, 34, 36] or at a practice and individual level [35]. Two studies proposed different statistical models to use data as a performance indicator to benchmark surgical departments [29, 30]. Benchmarking was performed once [24, 27–30] or more frequently [34–36], and feedback was provided via web-based systems [27, 28, 34, 35], individual report cards [24, 36] or via a peer-reviewed study [29, 30]. When individual healthcare professionals were benchmarked, most studies used adjusted outcome information to provide fair comparisons between individual healthcare professionals [28–30, 34–36]. In addition to benchmarked feedback, two studies also provided individual healthcare professionals with educational support [24, 28]. Four of eight studies reported on the impact of benchmarking, all showing no clinical effect.

### PDSA cycle

Two studies used a PDSA cycle to improve the quality of care [32, 33]. Van Veghel et al. (2014) reported on the establishment of an online transparent publication service for aggregated patient-relevant outcomes. Subsequently, these data enable benchmarking between Dutch heart centres to improve quality and efficiency. However, this study was not able to provide benchmarked patient-reported data due to a low response rate and a lack of data [33]. The study from Partridge et al. was a cross-sectional post-intervention study and compared their outcomes with a previously published report from the Health and Social Care Information Centre (HSCIC) from August 2011. A significant improvement in the Oxford Knee Score was found after changing the practice of care [32].

### Dashboard as a feedback tool

Two studies used a web-based dashboard as a feedback tool [25, 26]. In the study by van Zijl et al. (2021), feedback was available through graphical analysis of patient characteristics and PROMs for individual rhinoplastic surgeons. The purpose of this dashboard was to identify learning and improvement needs or provide data-driven motivation to change concepts or surgical techniques [25]. In Reilly et al., a dashboard was established to consistently measure the value of total hip and total knee arthroplasty by combining surgeon-weighted PROMs, clinical outcomes and direct costs [26]. Neither study reported on the impact of these methods.

### Aggregated statistical analysis

One study investigated how clinical outcome measures can be linked to PROMs and concluded that the following methods were most appropriate: (1) analysing the factors related to a good or poor patient-reported outcome, and (2) analysing the factors related to agreement or disagreement between clinical and patient-reported outcomes [31].

## Qualitative studies

### Study characteristics

Table 3 shows the study characteristics of the qualitative studies included in this research. All three studies comprised semi-structured interviews [10, 11, 37]. Interviews were conducted amongst experts from the United Kingdom [10, 11], US [11], Ireland [37], Sweden [10] and the Netherlands [11]. The study from Boyce et al. comprises the qualitative evaluation [37] of a randomized controlled trial, which is discussed in the quantitative section [28].

**Table 3:** Study characteristics of qualitative studies

References	Aim	Design	Setting
Boyce et al. [37]	To explore surgeons’ experiences of receiving peer-benchmarked PROMs feedback and to examine whether this information led to changes in their practice	Semi-structured interviews	Orthopaedic surgeons *N* = 11 (feedback arm from Boyce et al. [28])
Prodinger et al. [10]	To examine supporting and hindering factors relevant to integrating PROMs in selected health information systems tailored towards improving quality of care across the entire health system	Semi-structured interviews	Experts related to NHS, England (*N* = 7) and to SHPR and SKAR, Sweden (*N* = 3)
Van der Wees [11]	To inform policy-makers of prudent next steps for implementing patient-reported outcomes in clinical practice and performance measurement programmes in order to maximize their impact on the quality of care	Semi-structured interviews	Clinical practitioners, measure developers, and leaders of performance measurement programmes *N* = 58 from 37 organizations United States, United Kingdom and the Netherlands

*NHS* National Health Service, *SHPR* Swedish Hip Arthroplasty Register, *SKAR* Swedish Knee Arthroplasty Register

### Barriers, facilitators and lessons learned

In the qualitative analysis, barriers, facilitators and lessons learned/neutral statements were derived and were grouped into the following three themes: (1) conceptual, (2) methodological and (3) practical (Table 4). The overview and description of the themes (i.e. codebook) with the occurrence of facilitators, barriers and lessons learned can be found in Table 4. The most important lessons learned for future implementation and research can be found in Table 5.

**Table 4:** Codebook: Facilitators (F), barriers (B) and neutral statements (N) per qualitative theme

Theme	Conceptual	Boyce [37]	Prodinger [10]	Van der Wees [11]
Stakeholders	Any statements about the engagement of stakeholders at the meso and macro level in order to succeed	B/F	B/F/N	F/N
Subjectivity of PROMs	Any statements indicating that PROMs are subjective measures, and patients are not able to distinguish between consequences and complications of treatment	B	B	–
Aligning PROMs with clinical data	Statements concerning the discrepancy between PROMs and clinical outcome	B		–
PROMs versus PREMs	Any statements indicating that clinicians (consultants) did not distinguish the difference between PROMS and measures of patient satisfaction or experience	B	–	–

Methodological				
Data collection				
Choice of measure	Any statements indicating the choice of measure, such as type of measurement (generic vs disease-specific), length of measurement, reliability and validity of measurement	B	B/F/N	B/N
Timing of data collection	Any statements indicating the timing of data collection and how this would influence performance ranking at different time points	B	–	–
Response rate of measurement	Any statements indicating the response rate from patients, for example short-term follow-up (high response rate), while using the collection of longitudinal data with repeated measures (low response rate). Clinician discusses results with patients (high response rate even though long-term follow up)	–	B	B
Focus of measurement	Any statements indicating the importance of focusing on this measurement within this field, such as clinical value of expected improvements in outcome and variability between professionals	B	–	–
Data processing				
Representativeness of collected data	Any statements concerning representativeness of the data when using PROMs for quality improvement strategies. On the one hand, related to patients, this includes selection bias, inadequate answers, health literacy and nonresponse. On the other hand, related to healthcare professionals, these include selection and treatment bias, comparison between healthcare professionals, and confidentiality of reporting	B	B	B/N
Responsibility of healthcare professionals	Any statements concerning being held responsible for outcome data and its consequences	B	B	B/N
Inadequate case-mix control	Any statements concerning the use of case mix and effect on making comparisons between professionals	–	B	B/N
Interpretation of feedback	Any statements about the (mis)interpretation of feedback by experts, training for interpretation, or norm values for performance indicators	B/N	B	–

Practical				
Resources	Any statements indicating the infrastructure of data collection, such as availability or complexity of electronic data collection methods, or incorporation and use of resources for data collection in normal workflow/routine care related to additional workload for PROMs collection, interpretation and usage	B	B/F/N	B/F

**Table 5:** Lessons learned for future implementation and research

• Involve stakeholders from the very start and create a shared vision between stakeholders • Use generic and disease-specific patient-reported outcome measures • Ensure that PROMs are administered at the right time during the health process • Provide feedback on performance to individual healthcare professionals • Ensure that the data are representative and that the statistical analysis is comprehensible • Provide healthcare professionals with training for adequate interpretation of aggregated PROM data • Enable a good infrastructure for adequate data collection and analysis by trained and qualified staff	

#### (1) Conceptual

The following four themes were derived: stakeholders, subjectivity of PROMs, aligning PROMS with clinical data, and PROMs versus patient-reported experience measures (PREMs). One facilitator for success that was mentioned was the engagement and commitment of stakeholders at both the meso and macro levels from the beginning [10, 11, 37]. Champions can advocate the added value of collecting PROMs, and governance and political will can be decisive for its success and sustainability [10, 37]. Healthcare providers differ in their attitudes regarding the usage of PROMs for quality improvements; some advocate for sceptics [37]. As a start, small-scale projects with willing clinicians is recommended instead of teams with limited interest or readiness [11].

These advocates often need to convince other healthcare professionals due to concerns about the scientific properties of PROM measures, in particular the subjective characteristics of these measures. Thus, healthcare professionals have underlying doubt about the patient’s ability to answer PROM questionnaires [10, 37]. Furthermore, difficult-to-accept discrepancies between the PROM outcome and the clinical experience from healthcare professionals' point of view were found, since expectations were that these two outcome measures would align [37]. Moreover, Boyce et al. (2018) found that healthcare professionals were not able to distinguish the difference between PROMs and measures of PREMs [37].

#### (2) Methodological

Within this main theme, a distinction was made between data collection (2a) and data processing (2b).

#### (2a) Data collection

The following four themes were derived: choice of measure, timing of data collection, response rate of measurement and focus of measurement. Patient-reported measures should be selected cautiously to be appropriate for the targeted population [37], to ensure comparability and to prevent burdening the patient [10, 11]. The combination of generic and disease-specific measures was seen as feasible and complementary [10, 11, 37], especially since generic measures facilitate good comparison, but are less able to detect variation [10]. Moreover, standardization of time points for data collection is advocated, as timing may influence the results [10]. For example, outcomes were measured during short-term follow-up when patients were not fully recovered [37]. Furthermore, to obtain high response rates, it is important to discuss the results of PROMs with the patient during consultation, especially during long-term follow-up [11]. Another reported barrier concerned the clinical value of performance measurement for interventions in a field where small variability a priori could be expected [37].

#### (2b) Data processing

Four themes were derived: representativeness of collected data, responsibility of healthcare professionals, inadequate case-mix control and interpretation of feedback.

It was mentioned that some healthcare professionals mistrusted quality improvement measures based on aggregated PROMs. First, the representativeness of the data used for benchmarking or quality improvement was seen as a barrier. Healthcare professionals expressed concern that the data would not reflect practice, the individual practitioner or the population of patients [10, 11, 37]. Furthermore, some patient groups were identified as a possible source of information and recall bias, such as patients with low health literacy or those with comorbidities who might confuse problems from one condition with another [37]. Additionally, patients’ answers might be influenced by their care expectations, with the belief that this information is used to rate care, or by the need to justify their decision to have an operation [10, 37]. Additionally, healthcare professionals may be tempted to manipulate data to obtain good performance rates by recruiting patients who are more likely to have good outcomes (i.e. selection bias) [10, 11, 37]. Second, healthcare professionals were afraid to be held unfairly responsible for outcome data that could be biased by differences in resources across hospitals [37], differences in support services at the community level [37] or factors that occurred outside of their control [10, 11]. Third, healthcare professionals worried that inadequate case-mix control of confounders would bias comparisons of healthcare providers. In addition, the lack of transparency of the statistical analysis made it difficult to engage with the data. Two solutions were provided to address these barriers: (1) only providing aggregated data collection for quality improvement at a very generic level, or (2) presenting results stratified into subgroups instead of risk- or case-mix adjustment [11]. Furthermore, healthcare professionals expressed difficulty in understanding the data, a lack of norms for good or poor performance [11], and a need for training or guided sessions to correctly interpret the aggregated PROM data [10, 37]. Quality improvement reports were able to identify how hospitals and healthcare professionals stand relative to one another, but they are often general and lack the ability to identify opportunities for real quality improvement or action [10], which is key for clinicians in engaging with data and processes [11].

#### (3) Practical

Statements related to practical implementation were grouped under “practical”.

One theme, resources, was derived. Funding to get the programmes started was seen as a key facilitator for further development in structural embedding in routine care. Overall, commitment and support from the government and healthcare organizations were seen as facilitators [10, 37]. The availability of resources for routine data collection and monitoring without disruption of workflow or additional workload was seen as important [10, 11, 37]. For example, the need for sufficient IT capacity and software to analyse the data enabled the data to be available quickly for healthcare professionals [10, 11, 37]. Additionally, the availability of tablets and assistance in the waiting room for completing questionnaires, the establishment of infrastructure for developing and disseminating annual reports [10], and the opportunity for data linkage and integration in hospital records were mentioned.

## Discussion

The aim of this mixed-methods systematic review was to describe and investigate the experience and effectiveness of quality improvement methods based on aggregated PROMs. Four quality improvement methods were identified, including benchmarking, PDSA cycles, web-based dashboards as feedback tools, and the provision of aggregated statistical analysis reports. In total, 13 quantitative and three qualitative studies revealed that there is limited empirical evidence concerning quality improvements based on aggregated use of available PROMs. Only five studies reported on the effectiveness of the applied quality improvement method, and only one descriptive study reported a significant improvement of PROMs after implementation of aggregated PROM feedback. The qualitative studies identified that the belief of stakeholders, the use of generic and disease-specific PROMs, and the availability of funding and resources were important facilitators for success. One reported barrier was that sceptical healthcare professionals mistrusted the use of aggregated PROMs due to the subjectivity of PROMs and the contradictory results of PROMs and clinical outcomes. Furthermore, they were afraid to be held unfairly accountable for biased results as a result of case mix, differences in resources across hospitals, differences in support services at the community level or factors that occurred outside of their control. Lessons learned from the qualitative studies included creating shared stakeholder vision and that feedback on individual performance should be directed to individual healthcare professionals to learn from the outcomes of their own patients.

One quantitative study did find an effect of using aggregated PROMs in the PDSA cycle [32], and used specific facilitating factors to generate representative data, such as engagement of all stakeholders, the use of a combination of generic and disease-specific questionnaires, and obtainment of a high response rate. However, the results of this methodologically inferior cross-sectional post-intervention study should be interpreted cautiously.

Methodological and practical barriers were considered a reason for not finding an effect of benchmarking. Weingarten et al. suggested that no effect of peer-benchmarked feedback was found due to the *choice of measure,* since only one generic outcome measure (functional status) was used [24]. The themes timing of data collection and timing of feedback were mentioned as important barriers in the included quantitative studies as well; a follow-up measurement was taken too early after providing peer-benchmarked feedback [28], provision of feedback started too late in the study [34] or the authors mentioned that the duration of the intervention was too short to be fully adopted by all participating healthcare professionals [36]. Multiple studies had shortcomings in reporting on bias due to an insufficient response rate of the measurement. As PROMs are prone to missing data, it is important that studies adequately report on the completeness of data and take possible bias into account when drawing conclusions.

Another issue mentioned was the representativeness of the collected data, as some outcomes could not be linked to one specific surgeon, or low-volume surgeons were excluded from the analysis, which caused less variation [34]. Kumar et al. (2021) mentioned that the difficulty in feedback interpretation for healthcare professionals caused a lack of effect [36]. To improve understanding and interpretation, the use of training (e.g. statistics and visualization) and educational interventions was mentioned explicitly within the two randomized controlled trials addressing the quality improvement method of peer-benchmarked feedback [24, 28]. The importance of training was also addressed by the qualitative findings [10, 11, 37]. Previous research indicates that educational support is an important contextual factor for success in quality improvement strategies [38].

Additionally, the importance of good *resources* was mentioned in the discussion of the quantitative studies [24, 28, 34]. The importance of structural implementation was underlined by Varagunam et al. (2014), who stated that the small effect of the national PROMs programme was partly caused by the delay in the representation of the collected data.

### Strengths and limitations

A major strength of this review is the mixed-methods design with the inclusion of overall moderate- to good-quality studies, which enabled a comprehensive overview of all available quantitative and qualitative research within this field. Furthermore, due to the mixed-methods design of this review, the quantitative findings were discussed in light of the derived qualitative barriers, facilitators and lessons learned. As a result of the lack of empirical research concerning quality improvement methods based on the aggregated use of PROMS, a meta-analysis was not performed. Additionally, it was purposively decided to include only peer-reviewed studies, and it is acknowledged that important studies from the grey literature may have been missed.

### Future perspective

Future implementation of aggregated PROM feedback can be substantiated with the reported facilitators, barriers and lessons learned from the current review (Tables 4, 5). It is important that every institution using aggregated PROMs make their results available, including possible biases and completeness of outcome data. Furthermore, the strength of combining PROMs, clinical data and PREMs should be recognized. The use of aggregated clinical data and PREMs has already been shown to be effective in quality improvement [5, 39–41], while using aggregated PROMs for quality improvement is still in its infancy.

As qualitative outcomes mainly addressed the issue of obtaining accurate data and consequently gaining professionals’ trust in the concept and relevance of quality improvement, this research did not find best practices on how to learn and improve based on aggregated PROM data. Future research should focus on organizational and individual aspects that contribute to the optimal use of the obtained aggregated PROMs for quality improvement [42].

## Conclusion

This review synthesized the evidence on the methods used and effectiveness for quality improvement in healthcare based on PROMs. The findings demonstrate that four quality improvement methods are used: benchmarking, PSDA cycles, dashboards and aggregated analysis. These methods showed little to no effect, which may be due to methodological flaws, as indicated by the qualitative results. In conclusion, this field of research is in its infancy, and more empirical research is needed. However, the descriptive and effectiveness findings provide useful information for the future implementation of value-based healthcare at the meso level and further quality improvement research. In future studies, it is important that a shared stakeholder vision is created, PROMs and timing of measurement and feedback are appropriately chosen, interpretation of the feedback is optimal, every effort is made to reduce missing data, and finally, practical resources for data collection and feedback infrastructure are available.

## Supplementary Information

**Additional file 1:** **Appendix I**: Search strategy.

**Additional file 2:** **Appendix II**: Quality appraisal of included studies.

## Data Availability

Data collection forms and Rayyan extraction files can be obtained on request.
