# Supplementary material for: Quality improvements of healthcare trajectories by learning from aggregated patient-reported outcomes: a mixed-methods systematic literature review
Source: Health Res Policy Syst. 2022 Aug 17;20:90. doi: 10.1186/s12961-022-00893-4 (PMC9387033; doi:10.1186/s12961-022-00893-4)
Supplement: Supplementary file 1 — Additional file 1: Appendix I: Search strategy. [file 12961_2022_893_MOESM1_ESM.docx]

**Appendix I. Search strategy

Embase.com**

('patient-reported outcome'/mj/de OR (('questionnaire'/mj/exp OR 'self report'/mj/de OR 'patient satisfaction'/mj OR 'patient preference'/mj OR 'personal experience'/mj) AND ('quality of life'/mj/de)) OR (((patient* OR client* OR self) NEAR/3 (report* OR satisf* OR prefer* OR priorit* OR voice* OR centre* OR center* OR experience* OR assess*) NEAR/6 (outcome* OR measure* OR assess* OR quality-of-life)) OR ((questionnaire*) NEAR/6 (outcome* OR measure* OR assess*)) OR proms OR prom OR pros OR prem OR prems):ti) AND ('total quality management'/mj/de OR 'health care quality'/mj OR benchmarking/mj OR ('clinical effectiveness'/mj/exp AND ('program evaluation'/exp OR 'evaluation study'/exp)) OR 'performance measurement system'/mj OR 'safety'/de OR 'patient safety'/de OR (((benefit* OR advantage* OR disadvantage* OR effectiveness OR efficac* OR quality* OR impact* OR improv* OR evaluat* OR enhanc*) NEAR/3 (care OR healthcare OR communicat* OR decision-mak* OR practice*)) OR (((quality* OR performance*) NEAR/3 (impact* OR improv* OR Measure* OR indicator*)) NOT quality-of-life) OR (evaluat* NEAR/3 method*) OR benchmarking):ti) NOT ([conference abstract]/lim AND [1800-2018]/py)

**Medline ALL Ovid**

(*Patient Reported Outcome Measures/ OR ((*"Surveys and Questionnaires "/ OR *Self Report/ OR *Patient Satisfaction/ OR *Patient Preference/) AND (*Quality of Life/)) OR (((patient* OR client* OR self) ADJ3 (report* OR satisf* OR prefer* OR priorit* OR voice* OR centre* OR center* OR experience* OR assess*) ADJ6 (outcome* OR measure* OR assess* OR quality-of-life)) OR ((questionnaire*) ADJ6 (outcome* OR measure* OR assess*)) OR proms OR prom OR pros OR prem OR prems).ti.) AND (*Total Quality Management/ OR *Quality of Health Care/ OR *Benchmarking/ OR (*Treatment Outcome/ AND (*Program Evaluation/ OR *Evaluation Study/)) OR Safety/ OR Patient Safety/ OR (((benefit* OR advantage* OR disadvantage* OR effectiveness OR efficac* OR quality* OR impact* OR improv* OR evaluat* OR enhanc*) ADJ3 (care OR healthcare OR communicat* OR decision-mak* OR practice*)) OR (((quality* OR performance*) ADJ3 (impact* OR improv* OR Measure* OR indicator*)) NOT quality-of-life) OR (evaluat* ADJ3 method*) OR benchmarking).ti.)

**Cochrance CENTRAL register of trials**

((((patient* OR client* OR self) NEAR/3 (report* OR satisf* OR prefer* OR priorit* OR voice* OR centre* OR center* OR experience* OR assess*) NEAR/6 (outcome* OR measure* OR assess* OR quality-of-life)) OR ((questionnaire*) NEAR/6 (outcome* OR measure* OR assess*)) OR proms OR prom OR pros OR prem OR prems):ti) AND ((((benefit* OR advantage* OR disadvantage* OR effectiveness OR efficac* OR quality* OR impact* OR improv* OR evaluat* OR enhanc*) NEAR/3 (care OR healthcare OR communicat* OR decision-mak* OR practice*)) OR (((quality* OR performance*) NEAR/3 (impact* OR improv* OR Measure* OR indicator*)) NOT quality-of-life) OR (evaluat* NEAR/3 method*) OR benchmarking):ti)

**CINAHL EBSCOhost**

(MM Patient-Reported Outcomes OR ((MM " Questionnaires" OR MM Self Report OR MM Patient Satisfaction OR MM Patient Preference) AND (MM Quality of Life)) OR TI(((patient* OR client* OR self) N2 (report* OR satisf* OR prefer* OR priorit* OR voice* OR centre* OR center* OR experience*) N5 (outcome* OR measure* OR assess*)) OR ((questionnaire*) N5 (outcome* OR measure* OR assess*)) OR proms OR prom OR pros OR prem OR prems)) AND (MM Quality Improvement OR MM Quality of Health Care OR MM Benchmarking OR (MM Clinical Effectiveness AND (MM Program Evaluation OR MM Evaluation Research)) OR TI(((benefit* OR advantage* OR disadvantage* OR effectiveness OR efficac* OR quality* OR impact* OR improv* OR evaluat* OR enhanc*) N2 (care OR healthcare OR communicat* OR decision-mak* OR practice*)) OR (((quality* OR performance*) N2 (impact* OR improv* OR Measure* OR indicator*)) NOT quality-of-life) OR (evaluat* N2 method*) OR benchmarking))
