# Supplementary material for: Quality improvements of healthcare trajectories by learning from aggregated patient-reported outcomes: a mixed-methods systematic literature review
Source: Health Res Policy Syst. 2022 Aug 17;20:90. doi: 10.1186/s12961-022-00893-4 (PMC9387033; doi:10.1186/s12961-022-00893-4)
Supplement: Supplementary file 2 — Additional file 2: Appendix II: Quality appraisal of included studies. [file 12961_2022_893_MOESM2_ESM.pdf]

		Boyce, 2015[28]	Weingarten, 2000[24]	Kumar, 2021[36]	Varagunam, 2014[34]	Bronserud, 2019[29]	Van zijl, 2021[25]	Lucas, 2017[27]	Gutacker, 2013[30]	van Veghel, 2016[33]	Lundstrom, 2013[31]	Zheng, 2014[35]	Partridge, 2016[32]	Boyce, 2018[37]	van der Wees, 2014[11]	Prodinger, 2018[10]
**Randomized studies**	Is randomization appropriatly performed?	Yes	Yes													
	Are the groups comparable at baseline?	Yes	Yes													
	Are there complete outcome data?	Yes	No													
	Are outcome assessors blinded to the intervention provided?	No	No													
	Did the participants adhere to the assigned intervention?	Yes	Yes													
**Non-randomized studies**	Are the participants representative of the target population?			Yes	Yes	Yes	Yes	Yes	Yes	Yes						
	Are measurements appropriate regarding both the outcome and intervention (or exporsure)?			Yes	Yes	Yes	yes	Yes	Yes	Yes						
	Are there complete outcome data?			Yes	Can't tell	No	Yes	yes	Can't tell	No						
	Are the confounders accounted for in the design and analysis?			Yes	Yes	Yes	Can't tell	No	Yes	Can't tell						
	During the study periode, is the intervention administered (or exposure occurred) as intended?			Yes	Yes	Yes	Yes	Yes	Yes	Yes						
**Descriptive studies**	Is the sampling strategy relevant to address the research question?										Yes	Yes	Yes			
	Is the sample representative of the target population?										Yes	Yes	Yes			
	Are the measurements appropriate?										Yes	Yes	Yes			
	Is the risk of nonresponse bias low?										Can't tell	Can't tell	Yes			
	Is the statistical analysis appropriate to answer the research question?										Yes	Can't tell	Yes			
**Qualitative**	Is the qualitative approach appropriate to answer the research question?													Yes	Yes	Yes
	Are the qualitative data collection methods adequate to address the research question?													Yes	Yes	Yes
	Are the findings adequately derived from the data?													Yes	Yes	Yes
	Is the interpretation of results sufficiently substantiated by data?													Yes	Yes	Yes
	Is there coherence between qualitative data sources, colelction, analysis and interpretation?													Yes	Yes	Yes
